# Minimally invasive surgical treatments for male lower urinary tract symptoms/benign prostatic enlargement: A review on sexual function outcomes

**DOI:** 10.14440/bladder.2025.0001

**Published:** 2025-06-11

**Authors:** Eric Chung

**Affiliations:** 1 Department of Urology, Princess Alexandra Hospital, University of Queensland, Brisbane, Queensland 4102, Australia; 2 AndroUrology Centre, Brisbane, Queensland 4000, Australia; 3 Department of Urology, Macquarie University Hospital, Sydney, New South Wales 2113, Australia

**Keywords:** Benign prostatic hyperplasia, Lower urinary tract symptoms, Minimally invasive surgery, Sexual function, Erectile dysfunction, Ejaculatory function, Clinical outcomes

## Abstract

**Background:**

Male lower urinary tract symptoms (LUTS) secondary to benign prostatic enlargement (BPE) represent a common urological condition, especially in older males. Literature supports a strong association between male LUTS/BPE and sexual dysfunction. While transurethral resection of the prostate is often considered the standard for male LUTS/BPE surgical treatment, it causes complications across various male sexual domains, such as erectile dysfunction and ejaculatory disorders. In recent years, there have been considerable advances in minimally invasive surgical treatments (MISTs) for male LUTS/BPE. Published literature has shown that MISTs can improve various parameters of voiding domains while minimizing adverse sexual effects.

**Objective:**

This article provides an overview of the present understanding of the association between male LUTS/BPE and sexual dysfunction, and the present commercially available MISTs, with a specific focus on sexual function outcomes.

**Conclusion:**

Present MISTs for male LUTS/BPE have demonstrated considerable improvements in various urinary parameters while minimizing adverse effects across different sexual function domains.

## 1. Introduction

Male lower urinary tract symptoms (LUTS) secondary to benign prostatic enlargement (BPE) are a common condition affecting males. Globally, there were 94 million prevalent cases of BPE in 2019, compared= to 51.1 million cases in 2000.^1^ While it is accepted that male LUTS/BPE will become more common with age, metabolic risk factors, such as hypertension, dyslipidemia, diabetes, and obesity, have been shown to have a strong correlation with the development and subsequent progression of this condition.^2^,^3^ These risk factors also increase the risk of male sexual dysfunction, specifically erectile dysfunction (ED). Published literature has shown a link between male LUTS/BPE and ED through complex pathophysiological mechanisms such as the nitric oxide-cyclic guanosine monophosphate and RhoA/Rho-kinase pathways, autonomic hyperactivity, pelvic blood flow alteration, sex hormone imbalance, and chronic inflammation.^4^

The present management of male LUTS/BPE focuses on modifications of dietary and behavioral factors, optimization of underlying medical comorbidities, and commencement of medical therapy such as alpha-blockers and/or 5-alpha reductase inhibitors.^5^-^7^ Surgical intervention is traditionally reserved for cases of medically refractory patients or those who develop complications from BPE, such as recurrent hematuria, urinary tract infections, bladder stone(s), or obstructive uropathy.^6^-^9^ The transurethral resection of the prostate gland (TURP) is often considered the standard of comparison for all BPE surgical treatments and remains the most common surgery for male LUTS/BPE in Australia,^10^ but it can have significant complications, specifically in various male sexual domains such as erectile and ejaculatory problems.

Both ED and ejaculatory dysfunction (EjD) are known side effects of medications and TURP.^11^-^13^ Recently, there has been greater awareness and increasing emphasis by patients to preserve sexual function when choosing the appropriate BPE treatment.^14^ Hence, there is a strong interest among urologists and patients to seek out effective yet minimally invasive surgical treatments (MISTs) for male LUTS/BPE that can be performed as day surgery, have robust clinical outcomes, and are associated with minimal adverse impact across various sexual function domains.^15^ This article provides an overview of the preent understanding of the association between male LUTS/BPE and sexual dysfunction, and the present commercially available MISTs, with a specific focus on sexual function outcomes.

## 2. Search strategy

This study offers an overview of established and commercially available MIST for male LUTS/BPE in Australia. A Medline literature search for English language papers using the keywords “benign prostatic hyperplasia,” “lower urinary tract symptoms,” “minimally invasive surgery,” “sexual function,” “erectile dysfunction,” “ejaculatory function,” and “clinical outcomes” was performed and summarized in this narrative review. Given the broad scope of this review paper and the constraints of the author’s guidelines, an emphasis is placed on recent, high-quality references of scientific relevance, rather than adopting a full Preferred Reporting Items for Systematic Reviews and Meta-Analyses protocol.

## 3. Minimally invasive surgical techniques for male LUTSs/BPE

**Figure 1:**
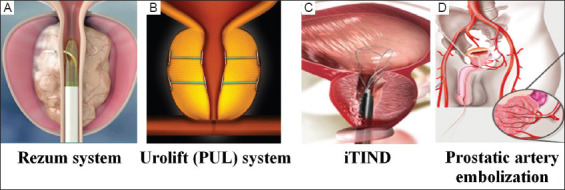
Minimally invasive surgical treatments for male lower urinary tract symptoms/benign prostatic enlargement. Images adapted from (A) Rezum (public domain image from Boston Scientific), (B) Urolift (PUL) system (public domain image from Teleflex), (C) ITIND (public domain image from Olympus), (D) Prostate artery embolization (generic Google image). Abbreviations: iTIND: i-Temporary implantable nitinol device; PUL: Prostatic urethral lift.

The present commercially available MISTs (Figure 1) for male LUTS/BPE can be largely divided into two categories: endourological and endovascular. Endourological interventions can be either prostatic ablative or non-ablative technology, while prostatic artery embolization (PAE) constitutes the main endovascular approach in the treatment of male LUTS/BPE.^14^ Table 1 presents the MISTs for male LUTS/BPE with no known or limited adverse effects on sexual outcomes.

**Table 1:** Minimally invasive surgical treatments for male lower urinary tract symptoms/benign prostatic enlargement

Benign prostatic hyperplasia treatment	Mechanism of action	Erectile function	Ejaculatory function
Rezum system	Convective water vapor energy therapy delivered by prostatic injection to ablate prostate tissue	None	Minimal
Urolift system (prostatic urethral lift)	Monofilament intraprostatic implant that compresses prostate tissue between a nitinol capsular tab and a urethral stainless-steel tab	None	None
i-Temporary implantable nitinol device	Temporary endoluminal struts with interlaced nitinol wires designed to cause delayed prostate tissue ischemia and necrosis	None	None
Prostatic artery embolization	Embolization of prostatic vessels to cause targeted ischemia and infarction	Minimal	Minimal

### 3.1. Endourological prostatic interventions

While conventional TURP resects prostate tissue to open the prostatic urethra and bladder outlet, these novel MISTs either expand the prostatic tissue or cause delayed prostatic tissue cavitation.

The inclusion criteria for these MISTs are often based on published pivotal clinical trials and adhering to the manufacturer’s guidelines. These include prostate volume <80 g and absence of (obstructive) median lobe or infection (such as prostatitis or urinary tract infection). The exclusion criteria include a history of detrusor overactivity, prior history of prostate surgery, radiation therapy, pelvic trauma, or neurological disease, and those on anticoagulants or antiplatelets.

### 3.2. Prostatic ablation: Rezum

The Rezum system (Boston Scientific, USA) is a transurethral water vapor therapy that utilizes thermal energy to create localized tissue necrosis and cavitation of the prostate gland over weeks and months.^16^,^17^ This system comprises a radiofrequency generator with a single-use transurethral delivery device that delivers thermal energy through a retractable vapor needle, in the form of water vaporization to induce cellular necrosis. Each injection process takes <10 s, and the number of injections required is dependent on the size of the prostate gland. Patients will need an indwelling catheter post-operatively, and the length of catheterization is largely based on the number of injections performed on the prostate gland.^17^

Published literature on the Rezum system has shown that this ablative device is effective in improving urinary symptoms and flow rate.^18^,^19^ This clinical improvement appears to past up to 4 years in intermediate-term studies, and the re-operation rate for Rezum at 4 years was 4.4%.^20^ While *de novo* ED has not been reported in clinical trials, up to 4% of patients who underwent Rezum therapy complained of postoperative EjD.^21^ More common treatment-related adverse events of Rezum include dysuria (16.9%), hematuria (11.8%), frequency and urgency (5.9%), acute urinary retention (3.7%), and urinary tract infection (3.7%).^18^-^20^,^22^-^24^

### 3.3. Prostatic non-ablative technology

In contrast to ablative technology, which creates prostatic tissue cavitation, these non-ablative surgical interventions relieve prostatic obstruction through mechanical distraction, either in the form of an intraprostatic implant or an endoluminal stent.

#### 3.3.1. Prostatic urethral lift (Urolift)

Prostatic urethral lift (PUL), also known as the Urolift device (Neotract Inc., USA), is a custom-designed disposable cartridge system that delivers a unique permanent monofilament implant. The implant consists of a nitinol capsular tab and a urethral stainless-steel tab bridged in between by a non-absorbable polyethylene terephthalate monofilament suture. The device applies tension by compressing the urethral surface against the prostatic capsule and cutting the tensioned suture to complete the deployment within the prostatic tissue. The present model provides one implant per cartridge system, with the number of implants needed to provide displacement of the prostatic lobes dependent on the adenoma size and configuration.^25^ The delivery of the implant compresses the prostatic tissue, resulting in focal ischemia and atrophy. Over the past decade, advances in surgical techniques have been described to deal with cases of the high bladder neck and prostatic middle lobe.

It has been almost a decade since the phase I trial was undertaken in Australia, and over the intervening years, there have been numerous clinical trials comparing PUL and TURP, real-world data, and systematic reviews,^26^-^33^ showing that PUL significantly improves various urinary parameters. Notably, no ED or EjD has been reported to date. Clinical data showed reasonable mechanical durability beyond five years, and a recent systematic review and meta-analysis concluded that the annual rate of surgical re-intervention following PUL was around 6.0% per year.^30^

#### 3.3.2. i-Temporary implantable nitinol device

The i-Temporary implantable nitinol device (iTIND) (Medi-Tate Ltd, Israel) is the second-generation version of the TIND device that consists of three elongated struts organized with interlaced nitinol wires at 12, 5, and 7 o’clock positions with an open-ended tip and a nylon wire to attach to the anchoring leaflet.^34^ Once the device is deployed, the plastic sheath covering the nylon wire can be taken off, and the wire can be shortened. The device is left *in situ* for 5 – 7 days to achieve maximal strut expansion and radial compression of the prostatic tissue. The nylon wire anchored to the device coming out of the urethral meatus can be removed by direct vision using a cystoscopy to close the device or retrieved through an open-ended catheter.^35^ The struts determine a circumferential force producing ischemia and necrosis of the mucosa, creating prostatic incisions at 12, five, and seven o’clock positions to open the bladder outlet.^36^ As the newest MIST, there are fewer published studies on i-TIND. Nonetheless, this device has improved both urinary flow rate and function.^37^-^40^ Similarly, no ED or EjD has been reported to date.^37^,^39^

### 3.4. Endovascular prostatic intervention: PAE

While PAE was first developed to control major prostatic bleeding, its role in treating male LUTS/BPE has taken stronger prominence in the past few years.^41^-^44^ Patients are required to undergo appropriate computed tomography angiography to delineate prostatic vascular anatomy since variations and frequent anastomoses can present technical challenges during the procedure.^45^,^46^ After an arterial puncture (either common femoral artery or radial artery), the internal iliac (or hypogastric) artery is cannulated, followed by super-selective catheterization of the prostatic artery to deliver various embolic materials such as metallic coils, microspheres, gelatin sponge (Gelfoam), trisacryl gelatin microspheres, or polyvinyl alcohol particles.^45^-^47^ Recent refinements in the technical aspects of PAE include cone-beam computed tomography capability, embolization techniques, as well as optimization of the types and sizes of embolic agents to improve clinical outcomes.^45^-^47^ PAE achieves targeted ischemia and subsequent tissue infarction of the prostate with apoptosis and loss of the prostatic adenoma.

Published literature, including those with direct comparative trials against TURP as well as systematic reviews and meta-analyses, has shown that PAE can be effective and safe in a carefully selected group of men.^48^-^52^ While PAE offers a valid alternative for BPE treatment for improvements in urinary function and flow rate, non-targeted embolization to other organs, such as the bladder, penis, and rectum, can be a serious complication with subsequent ischemia and ulceration. Hence, there is still a risk of ED and EjD.^53^

## 4. Clinical recommendations on MISTs

To date, there is no well-conducted study exploring the cost-effectiveness of MISTs or evaluating actual patient decision-making in selecting a MIST over another. However, one study examined the six common benign prostatic hyperplasia therapies^54^ and found that combination prescription drug therapy was the least effective and provided one-third of the symptom relief achieved with MISTs. TURP and photoselective vaporization of the prostate provided slightly greater relief of LUTS than MISTs at approximately twice the cost over 2 years.

It is invariably that MISTs become the new standard of therapy for male LUTS/BPE, given the significant interest in this technology, particularly for their suitability as day procedures, faster recovery time, and preservation of sexual function domains (Table 2). In recent years, patients have become tech-savvy, more educated about their preferences, and are pushing for shared decision-making on the latest device and what is best for them. Given that the recent American Urology Association guidelines^5^,^8^ highlighted the potential of irreversible bladder damage with delayed benign prostatic hyperplasia treatment and placed greater emphasis on sexual function preservation, MIST should be considered for sexually active patients with LUTS/BPE. However, clinicians should adhere to the present limitations of MIST and offer it to the right surgical candidates based on the inclusion and exclusion criteria listed by each device manufacturer and published clinical trials.

**Table 2:** Key benefits of minimally invasive surgical treatments

Advantages of minimally invasive surgical treatments
Effective and durable symptomatic relief from lower urinary tract symptoms/benign prostatic enlargement
Preservation of all male sexual domains, including erectile and ejaculatory functions
Same-day procedure
Minimal anesthesia requirements
Quicker recovery and return to normal physical activity
Better adverse event profile than transurethral resection of the prostate (such as pain, bleeding, reoperation, and stricture) Surgical complication(s) is easily managed

## 5. Conclusion

Present and commercially available MISTs for male LUTS/BPE have demonstrated considerable improvements in various urinary parameters while minimizing adverse effects across different sexual function domains, such as ED and EjD. While MISTs will be considered the new standard of surgical care in the future, it is pivotal to ensure suitable patient selection, proper informed consent, and careful application of these promising BPE technologies for better clinical outcomes beyond those achieved by the present TURP standard. Further validation of the technical performance of these devices through direct, comparative multi-center trials and cost-analysis modeling is needed to determine their actual roles in the clinical management of male LUTS/BPE.

## Data Availability

Not applicable.
